# Waiting Mortuaries

**Published:** 1896-12-05

**Authors:** 


### Waiting Mortuaries.

It is gradually becoming recognised that in modern conditions of life our dealings with the dead require considerable modification, and in none more so than in the pernicious habit of retaining their bodies in the dwellings of the living for the long period which is at present customary. We are, however, in this matter on the horns of a dilemma. Sanitary science urges with considerable insistance that the dead body should be removed at the earliest possible opportunity, but, on the other hand, a vague uneasiness, a whispered dread of premature sepulture, urges people not to hasten too greatly the final irrevocable burial. Again and again this uneasiness finds voice—just as often to be drowned by the reiterated assertion, either that such a thing as being buried alive never happens, or that it occurs so rarely as not to be worthy of consideration. Regarded from the cold statistical point of view, much as railway directors calculate the probability of a given traveller meeting with a fatal accident, no doubt such an event is extremely rare. Yet there can be little doubt of its occasional occurrence, especially when, under the influence of panic during epidemics, the safeguards are lost which in ordinary times arise from the long period which in this country is allowed to intervene between death and burial.

Our interest in the matter is, however, sanitary rather than sentimental. We see a condition of society in which, from the heaviness of rent and the urgent necessity of living near their work, people are led to huddle together and live a very crowded life, and we cannot but recognise the justice of the sanitary demand that under such circumstances the dead should be removed as quickly as possible from the crowded dwellings of the living. On the other hand, we also see that if such a course meant early burial it might increase the risk, at present small, of premature sepulture, unless between removal from the dwelling and final burial or cremation, some intermediate resting-place were provided—not a dead-house of the present type, a mere warehouse for unclaimed bodies, but a waiting mortuary, a place in which the dead might be treated with reasonable decency, and might rest until the grave were ready and the fact of death declared itself beyond all cavil.

A book, which is reviewed in another column, has recently appeared on Premature Burial, and the authors appear to have ransacked pretty nearly all that has been published on the subject. There can be no doubt that the case as presented in its pages is one "to make your flesh creep." Many cases are related in which it is asserted that premature burial either has taken place, or has only been escaped by the merest accident. Among these may be specially noticed cases of trance, and of such diseases as cholera and small-pox, diseases which cause panic and lead to hasty interment. It must be confessed that we know but little about trance and those conditions of catalepsy with which it is allied. At anyrate we do not know to how low an ebb the functions of the body may be reduced under such conditions and yet be capable of resuscitation; and although we cannot admit that a complete medical examination would have failed to demonstrate the absence of death in the cases which are stated to have only escaped by apparent accident the dreadful fate of living burial, at the same time it must be remembered that under the present law no provision is made for medical inspection after death, real or supposed, and that the certificates on the strength of which bodies are buried, are issued on the mere statement of the most illiterate people. Thousands of people are buried every year without any certificate from any medical man, while even when a "medical certificate" is forthcoming, it is so worded as to certify only the presumed cause of death, but by no means to certify the fact of death having taken place. This is left to the informant, whoever he may be. The law takes considerable trouble to prevent a body being buried without being registered, but it takes no trouble whatever to prevent it being buried without being dead. This is left to the "common sense" of the relatives! And so long as bodies are kept some days above ground after death common sense will, doubtless, in the great majority of cases be right. We urge the provision of proper mortuaries, partly because such an arrangement will greatly facilitate early removal, partly because it will take away this constantly recurring scare of premature burial which to some people is a perfect nightmare.

